# Genome sequence of the brown rot fungal pathogen *Monilinia fructigena*

**DOI:** 10.1186/s13104-018-3854-z

**Published:** 2018-10-23

**Authors:** Lucia Landi, Rita M. De Miccolis Angelini, Stefania Pollastro, Domenico Abate, Francesco Faretra, Gianfranco Romanazzi

**Affiliations:** 1 Department of Agricultural, Food and Environmental Sciences, Marche Polytechnic University, Via Brecce Bianche 10, 60131 Ancona, Italy; 2 Department of Soil, Plant and Food Sciences, University of Bari ‘Aldo Moro’, Via G. Amendola 165/A, 70126 Bari, Italy

**Keywords:** Brown rot, De-novo assembly, Genome annotation, Illumina, *Monilinia fructigena*, PacBio, Pome fruit, Stone fruit

## Abstract

**Objectives:**

*Monilinia fructigena* (phylum *Ascomycota*, family *Sclerotiniaceae*) is a plant pathogen that causes brown rot and blossom blight in pome fruit and stone fruit of the *Rosaceae* family, which can cause significant losses in the field and mainly postharvest. The aim of this study was to create a high-quality draft of the *M. fructigena* genome assembly and annotation that provides better understanding of the epidemiology of the pathogen and its interactions with the host(s) and will thus improve brown rot management.

**Data description:**

We report here on the genome sequence of *M. fructigena* strain Mfrg269 that was collected from plum in southern Italy. This is assembled into 131 scaffolds, with a total size of 43.125 Mb, with 9960 unique protein-coding genes. The novel genomic resources allow improved genomic comparisons among the most important pathogens belonging to the *Monilinia* genus, with the aim being to improve the knowledge of their plant–pathogen interactions, population biology, and control.

## Objectives

*Monilinia fructigena*, Honey *ex* Whetzel is one of the several apothecial ascomycetes, which primarily include *Monilinia laxa* (Aderhold and Ruhland) Honey and *Monilinia fructicola* (G. Winter) Honey [1]. These can cause brown rot and blossom blight, which results in serious economic losses for crops of the *Rosaceae* family [2]. *M. fructigena* is widespread in Europe, Asia (e.g., Near East, Far East, India), northern Africa, and some parts of South America, and it is a quarantine pathogen in Canada, USA, Australia and New Zealand (http://www.cabi.org/isc/datasheet/34747). It causes one of the most important diseases on stone and pome fruit trees [3, 4]. Prevalent symptoms are fruit rots in the field and also postharvest.

The aim of this study was to provide new data on the sequence of the *M. fructigena* genome and the annotated protein-coding genes. Here, we report on the *M. fructigena* draft genome obtained using a hybrid assembly approach that exploited the high accuracy of the Illumina next generation sequencing along with the long-size of the Pacific Biosciences (PacBio) third generation sequencing, thus reducing gaps and improving the quality of the draft genome [5]. Our annotated genome draft is larger and of better quality than the publicly available *M. fructigena* genome [6]. Our data reduced the *M. fructigena* assembly to 131 scaffolds without gaps. In addition, the sequence completeness and high coverage were verified by mapping RNA sequencing (RNA-Seq) reads from the same *M. fructigena* strain [7]. The availability of a more accurate genome sequence provides improved opportunities to the scientific community for studies aimed at exploring in more detail the pathogen epidemiology, its host interactions, and the tools to optimise brown rot management.

## Data description

The data report here (Table 1) are related to the de-novo assembly and annotation of the genome of *M. fructigena*. A monoconidial strain of *M. fructigena*, Mfrg269, was derived from plum during monitoring of *Monilinia* populations present on stone fruit in southern Italy (Tursi, Basilicata) [8]. The strain was characterised both at the phenotypic and molecular levels [7–9]. The strain was grown in liquid medium (2% malt extract; Oxoid) for 36 h at 24 ± 1 °C, in darkness and under shaking (150 rpm). Genomic DNA was extracted using Gentra Puregene tissue kits (Qiagen, Milan, Italy), according to the manufacturer instructions. Genome sequencing yielded both short 2 × 92-bp paired-end reads (Illumina Sequencing Technology; HiScanSQ platform; SELGE Network Sequencing Service, Bari, Italy) (Table 1, dataset 1; Data Citation 1: National Center for Biotechnology Information (NCBI) Sequence Read Archive SRR7262862) and long 20-kb reads (PacBio Sequencing Technology; RSII platform; Macrogen Inc., Next Generation Sequencing Service, Geumcheon-gu, Seoul, South Korea) (Table 1, dataset 2; Data Citation 2: NCBI Sequence Read Archive SRR7263013). A hybrid assembly strategy was applied, and all of the reads from both platforms were assembled to produce scaffolds according to the DBG2OLC pipeline [5], with optimized parameters. SparseAssembler [10] was used to preassemble the Illumina short reads into contigs (NodeCovTh, 2; EdgeCovTh, 1; k, 71; g, 15; and PathCovTh, 100). The overlap and layout were performed with module DBG2OLC [5] with the output contig file and 20 × PacBio long reads (AdaptiveTh, 0.001; KmerCovTh, 2; and MiniOverlap, 20). The consensus was then performed using the Sparc module [11] in which BLASR [12] was used to align all of the raw reads to the assembly backbone, with the default settings. The selection of the best draft genome was also carried out by mapping RNA-Seq reads from the same *M. fructigena* strain [8] using the CLC Genomics Workbench v. 7.0.3 software (CLC Bio, Aarhus, Denmark). Gene prediction was performed with Augustus implemented in the BLAST2GO PRO package (v.4.1.9), using *Botrytis cinerea* as the model species and the RNA-Seq reads as a guide with default settings.

**Table 1:** Overview of data files

Label	Name of data file/dataset	File types (file extension)	Data repository: NCBI accession number
Dataset 1	SRR7262862 Illumina DNA sequencing of *Monilinia fructigena* strain Mfrg269	fastq/fasta files	https://www.ncbi.nlm.nih.gov/sra/SRX4167027
Dataset 2	SRR7263013 PacBio DNA sequencing of *Monilinia fructigena* strain Mfrg269	fastq/fasta files	https://www.ncbi.nlm.nih.gov/sra/SRX4167179
Data file 1	QKRW01000001-QKRW01000131 Whole genome shotgun sequencing of *Monilinia fructigena* strain Mfrg269	fasta/GenBank/ASN.1 files	https://www.ncbi.nlm.nih.gov/Traces/wgs/QKRW01

The result was a high-quality annotated gap-free draft genome (Table 1, data file 1; Data citation 3: GenBank QKRW01000001–QKRW01000131). About 83% of the RNA-Seq reads mapped on the final genome draft version. This had a total size of 43.125 Mb with 42.05% GC content, ~ 210× sequencing coverage, 131 scaffolds, N50 scaffold length of 767.732 kb, scaffold L50 of 20, and maximum scaffold size of 1,863,841 bp. The 10,502 genes, with 10,802 transcripts that coded for 9960 predicted proteins were functionally annotated on the draft genome.

The genome herein described is an improved version of the *M. fructigena* genome as compared to the previously published version obtained by next generation sequencing alone (GCA_002909635.1; genome length: 39.329 Mb; coverage: 110×; 1633 scaffolds; scaffold N50: 56.695 kb; L50: 208). These novel genomic resources make feasible better genomic comparisons among the most important pathogens belonging to the *Monilinia* genus, with the aim to improve knowledge of their phylogenic relationships, plant–pathogen interactions, population biology, and control.

## Limitations

These data report the genome sequence of a single strain of *M. fructigena* obtained by a hybrid approach using next generation sequencing and third generation sequencing and automatic gene prediction, although driven by RNA-Seq reads. An important further step will be the reconstruction of whole chromosomes and the manual curation of the predicted genes and their annotation for better characterization of the genome at both the structural and functional levels. Furthermore, studies on comparative genomics will be feasible when high quality genomic data of other *Monilinia* species are available.

## Abbreviations

NCBI: National Center for Biotechnology Information
PacBio: Pacific Biosciences
RNA-Seq: RNA sequencing
